# Lithium treatment and estimate glomerular filtration rate in bipolar disorder patients: A cross-sectional study

**DOI:** 10.1192/j.eurpsy.2021.236

**Published:** 2021-08-13

**Authors:** S. Fernandes, T. Cerqueira-Silva, A. Pinto, Â. Miranda-Scippa

**Affiliations:** Faculdade De Medicina Da Bahia, Universidade Federal da Bahia (UFBA), Salvador, Brazil

**Keywords:** bipolar disorder, serum lithium level, lithium, nephrotoxicity

## Abstract

**Introduction:**

Lithium has been the mainstay therapy for bipolar disorder (BD) for decades, but there is little consensus regarding its possible effects on kidney function and the rate of change in estimated glomerular flow rate (eGFR) over time.

**Objectives:**

To describe patients with BD regarding their renal function and their sociodemographic and clinical characteristics potentially related to eGFR.

**Methods:**

This is a cross-sectional study with an initial sample of 95 patients with BD. Multiple linear regression analysis was applied to investigate the association of lithium serum levels and their duration of treatment with eGFR, independent of confounding factors. We excluded patients without data regarding any of the variables from the final model.

**Results:**

In the multivariable analysis, the model was composed of eight variables (Figure 1). The mean duration of treatment was 10 years (Figure 2). Serum lithium level was associated with low levels of eGFR (β = -18.06 [-34.70 - -1.42], p = 0.03); among the other variables, only age remained associated with it (β = -0.72 (-1.10 - -0.33), p = <0.01).

**Figure:**
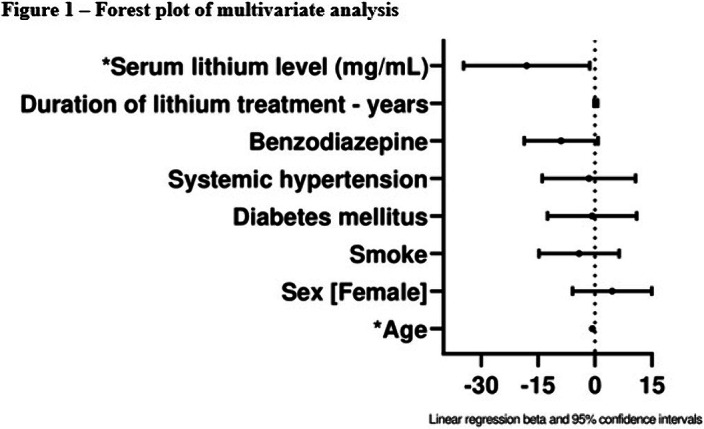
Note: *P<0.05

**Figure:**
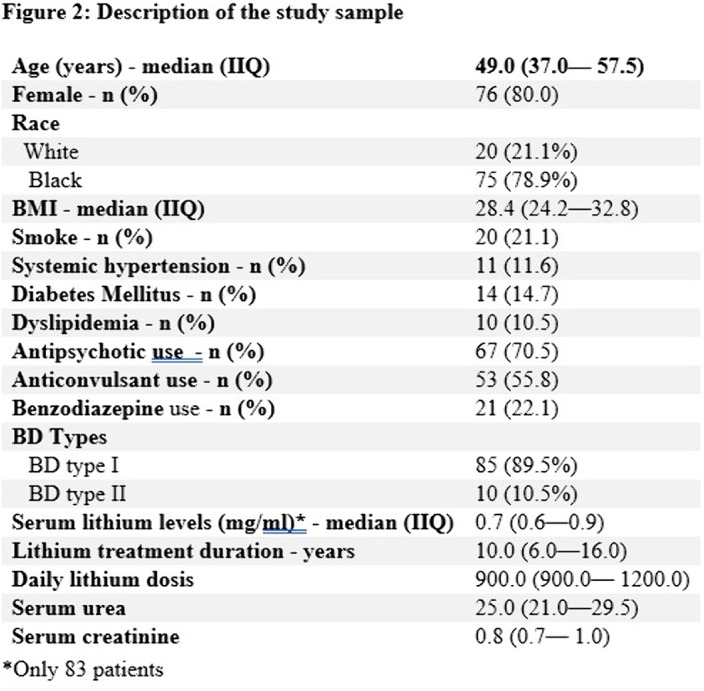

**Conclusions:**

We replicated the correlation between serum lithium levels and eGFR. Our results contradict the claim that duration of treatment with lithium correlates with lower levels of eGFR, while suggesting serum lithium level could be a possible early marker of lithium nephrotoxicity.

**Disclosure:**

No significant relationships.

